# Abdominal imaging in precocious puberty in girls: can imaging determine onset of puberty?

**DOI:** 10.1007/s00247-024-05992-8

**Published:** 2024-07-29

**Authors:** Anne M. Smets, Carmelo Sofia, Costanza Bruno, Damjana Ključevšek, Maria Luisa Lobo, Marcello Napolitano, H. Nursun Ozcan, Samuel Stafrace, Philippe Petit, Lil-Sofie Ording Müller

**Affiliations:** 1 Department of Radiology and Nuclear Medicine, Amsterdam UMC, University of Amsterdam, Meibergdreef 9, 1105 AZ Amsterdam, The Netherlands; 2 Department of Biomedical Sciences and Morphologic and Functional Imaging, University of Messina, Messina, Italy; 3 Radiology Department, AOUI Verona (Azienda Ospedaliera Universitaria Integrata), Verona, Italy; 4 Department of Radiology, University Children’s Hospital Ljubljana, Ljubljana, Slovenia; 5 Unidade Local de Saúde de Santa Maria (ULSSM, Former CHULN), Av Professor Egas Moniz, 1649-028 Lisbon, Portugal; 6 Department of Pediatric Radiology and Neuroradiology, V. Buzzi Children’s Hospital, Milan, Italy; 7 Department of Radiology/Division of Pediatric Radiology, Hacettepe University School of Medicine, Ankara, Turkey; 8 McMaster Children’s Hospital, McMaster University, Hamilton, Ontario Canada; 9 Department of Pediatric Radiology, Hôpital Timone Enfants, Aix Marseille-Université, Marseille, France; 10 Division of Radiology and Nuclear Medicine, Department of Paediatric Radiology, Oslo University Hospital, Oslo, Norway

**Keywords:** Child, Female, Ovary, Precocious, Puberty, Ultrasound, Uterus

## Abstract

Precocious puberty in girls is defined by the appearance of secondary sex characteristics before the age of 8 years. Precocious puberty is either peripheral or central. In most cases, the cause of central precocious puberty is unknown. Rapidly progressive forms of central precocious puberty may benefit from puberty suppression to prevent compromised final height and psychosocial problems related to the physical changes. Idiopathic central precocious puberty in girls can be a challenging diagnosis. Clinical examination, evaluation of growth charts, bone age, and hormonal tests are part of the workup. The role of pelvic ultrasound to evaluate pubertal features of the internal genitalia in this context is controversial. In this paper, we have reviewed the literature to find the scientific bases for several proposed ultrasound parameters and cutoff values for the determination of onset of puberty in girls with suspected central precocious puberty. There are no reliable cutoffs for the measurements of uterus and ovaries for the diagnosis of central precocious puberty and there is overlap in the appearance of the organs in both prepubertal and pubertal girls. The interpretation of the findings on pelvic ultrasound must be done with caution and always in close communication with the referring clinicians.

**Graphical Abstract:**

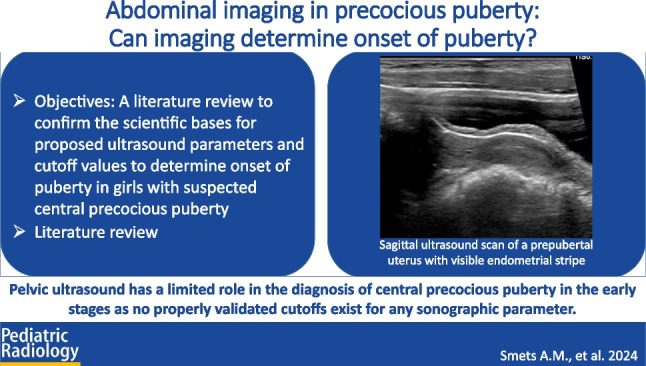

## Introduction

### Concepts in precocious puberty

Puberty is the biological transition between childhood and adulthood, characterized by hormonal, physical, and psychological changes. It is a complex process involving nutritional, genetic, ethnic, environmental, and geographic factors. In girls, the normal process of puberty starts with the activation of the pulsatile release of hypothalamic gonadotropin-releasing hormone (GnRH). GnRH activates the pituitary gland to release follicle stimulating hormone (FSH) and luteinizing hormone (LH). Increased levels of FSH and LH stimulate the ovaries: they grow and start to produce greater quantities of estradiol. The circulating estradiol stimulates the development of the secondary sexual characteristics such as the growth of the mammary glands and the uterus and it also accelerates bone maturation. In girls, puberty usually starts with the development of breast buds (thelarche), at a mean age of 10 years (range 8–12 years). The first periods (menarche) typically occur within 2–5 years after breast budding. Growth spurt is at a maximum before menarche. There is a wide variation in the age at onset and the speed of pubertal maturation. Furthermore, over the past few decades, there have been reports of a secular trend toward earlier pubertal onset: the age at thelarche seems to have decreased by 3 months per decade between 1977 and 2013 [1].

**Fig. 1:**
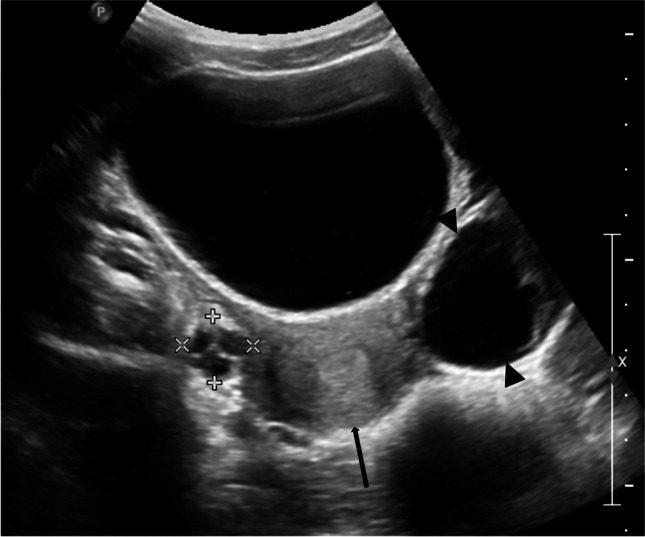
Axial gray-scale pelvic ultrasound image in a 5-year-old girl with McCune-Albright syndrome shows a large follicular cyst on the left ovary (*arrowheads*) and a stimulated uterus (*arrow*). The normal right ovary is marked by cursors

**Fig. 2:**
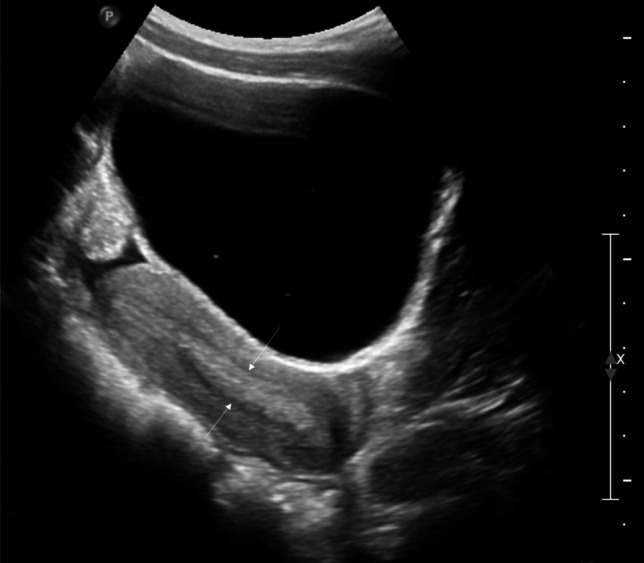
Sagittal gray-scale pelvic ultrasound in the same patient as in Fig. 1 shows a large stimulated uterus with a thick endometrium (*arrows*)

**Fig. 3:**
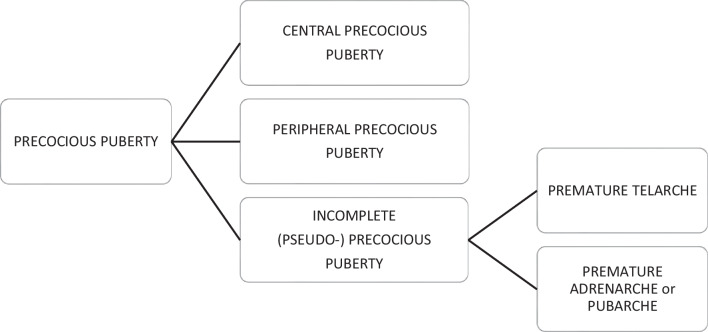
Types of precocious puberty

Precocious puberty in girls is defined by the appearance of secondary sex characteristics (breast budding and/or pubic and axillary hair) before the age of 8 years. GnRH-dependent or central precocious puberty is due to a premature activation of the hypothalamic–pituitary–gonadal (HPG) axis. It can be organic related to an intracranial lesion, or much more frequently idiopathic. In peripheral precocious puberty, the HPG axis is not activated. Isosexual peripheral precocious puberty is caused by the secretion of endogenous hormones from a functional follicular cyst, an ovarian tumor, in hypothyroidism, in McCune Albright syndrome, or by the exposure to exogenous hormones (Figs. 1 and 2) [2]. Prolonged peripheral precocious puberty can subsequently trigger early maturation of the HPG axis, resulting in progression from peripheral to central precocious puberty [3]. A third category encompasses forms of incomplete precocious puberty, namely isolated breast budding, i.e., premature thelarche, or isolated pubic or axillary hair development, i.e., premature adrenarche (Fig. 3).

The prevalence of idiopathic central precocious puberty in girls varies significantly in different populations from 10 to 200/100,000 girls per year [4–8]. This wide variation is probably due to the use of different definitions with or without inclusion of the incomplete forms. The latter group of patients requires to be identified because they may benefit from pubertal suppression. There are two peaks at which premature thelarche occurs: one around the age of 2 years, caused by a delay in inhibition of the transient physiological activation of the HPG axis also called the mini-puberty, and the second one between the ages of 6 years and 8 years. Isolated premature thelarche in 6–8-year-old girls can be seen in the context of obesity, which may cause adipomastia but also an increased peripheral estrogen production and true thelarche [9, 10]. However, in most cases, premature thelarche seems to be triggered by idiopathic mild overfunction of the HPG axis. Growth velocity is not accelerated in these girls and bone age is within normal ranges. Premature thelarche can also be a first sign of central precocious puberty. In clear-cut central precocious puberty, clinical examination shows true thelarche, accompanied by accelerated growth, advanced bone age, and a pubertal response of LH to the GnRH stimulation test.

### Diagnostic tests in precocious puberty

Discriminating between the common normal pubertal variants and true central precocious puberty, especially in the early stages, can be a diagnostic challenge. It is based on physical examination and growth velocity assessment, and the additional tests that can be used are bone age determination, hormonal tests, and pelvic ultrasound (US).

Bone age is often, but not always, advanced in precocious puberty and advanced bone age can also be found in overweight children and children with constitutional tall stature [11–13]. Hormonal analysis consists of the measurement of gonadotrophins and estradiol. The GnRH-stimulation test (GnRHST) is considered the gold standard for determining the onset of puberty and is indicated if the basal serum levels of LH and FSH are not diagnostic. The outcomes of the GnRHST can be ambiguous and cutoffs for diagnosis of central precocious puberty are not consistent [14, 15].

The development of the uterus and ovaries during childhood and adolescence has been extensively studied with US [16–33].

The role of abdominal imaging to look for the underlying cause of peripheral precocious puberty is well established. However, the value and meaning of abdominal imaging in determining onset of puberty is unclear. Since rapidly progressing central precocious puberty may cause psychological stress and early epiphyseal closure and hence a compromised final height, early diagnosis is essential for timely treatment with puberty blockers. Therefore, differentiating incomplete precocious puberty and slowly progressing central precocious puberty from the rapidly progressing form is essential. This topic has been researched extensively for decades but there is no consensus on US-based parameters and cutoffs.

In this paper, we present an overview of the physiological development of the internal genitalia and ovaries as a background to comprehend the findings in pelvic US. Thereafter, we present the results of an extensive literature review performed to find the scientific bases for several proposed US parameters and cutoff values for the determination of onset of puberty in girls with suspected central precocious puberty.

## Physiological development of the pelvic organs

### The uterus

**Fig. 4:**
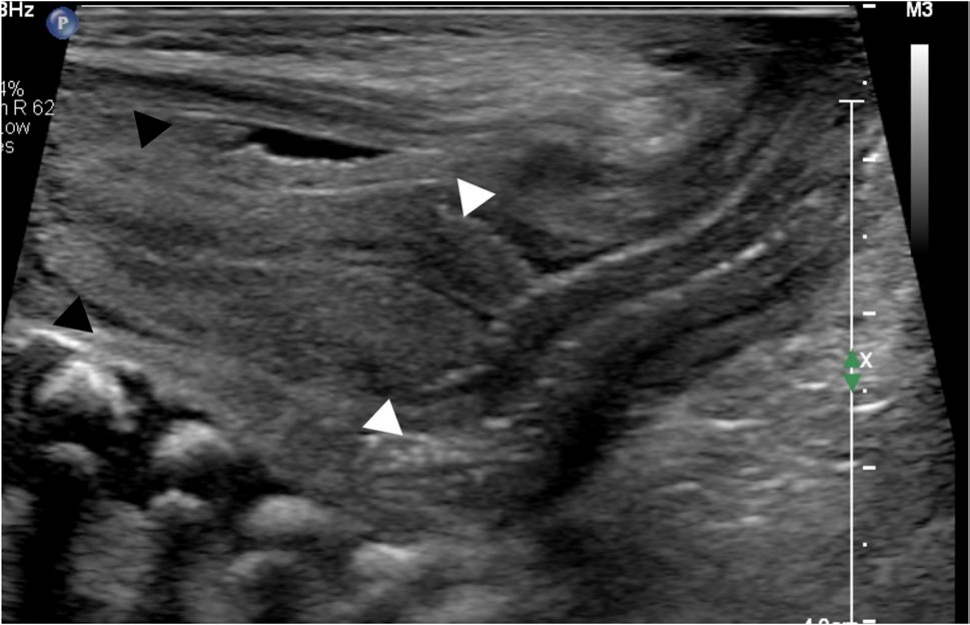
Sagittal gray-scale ultrasound image from a perineal approach in a female neonate shows a spade-shaped uterus, the cervix (*white arrowheads*) being wider than the fundus (*black arrowheads*)

**Fig. 5:**
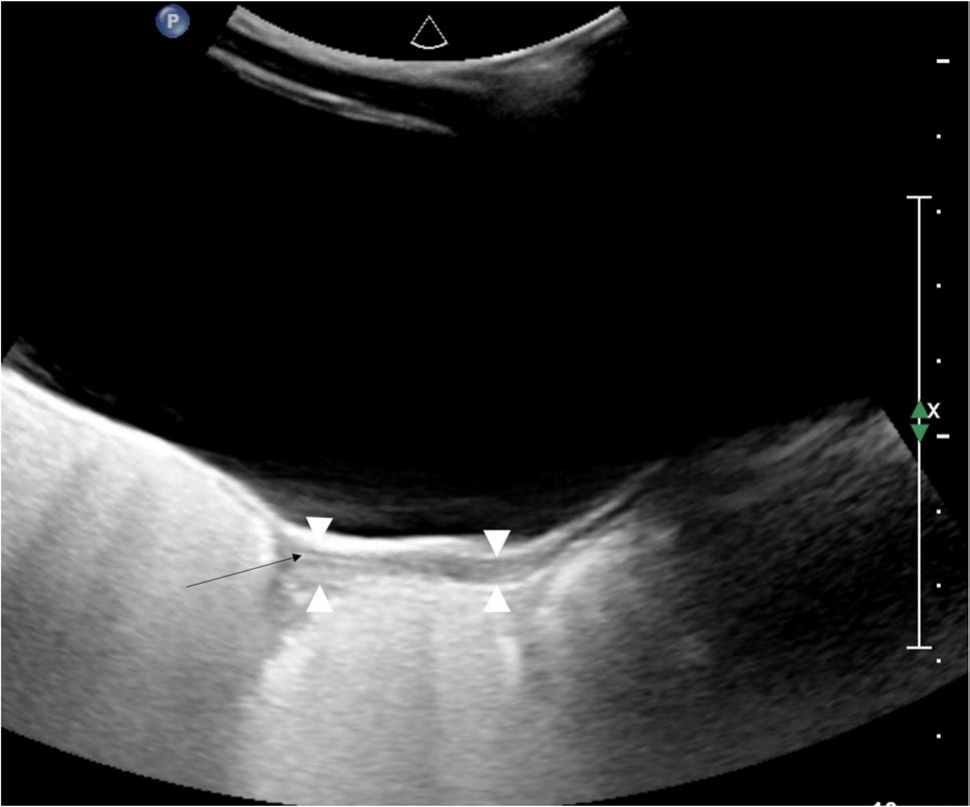
Prepubertal 8-year-old girl. Sagittal gray-scale pelvic ultrasound in an 8-year-old prepubertal girl shows a small tubular uterus in which the anteroposterior diameters of cervix and fundus (*arrowheads*) are equal*.* Cervix and fundus cannot be individualized. The endometrium is thin (*black arrow*)

**Fig. 6:**
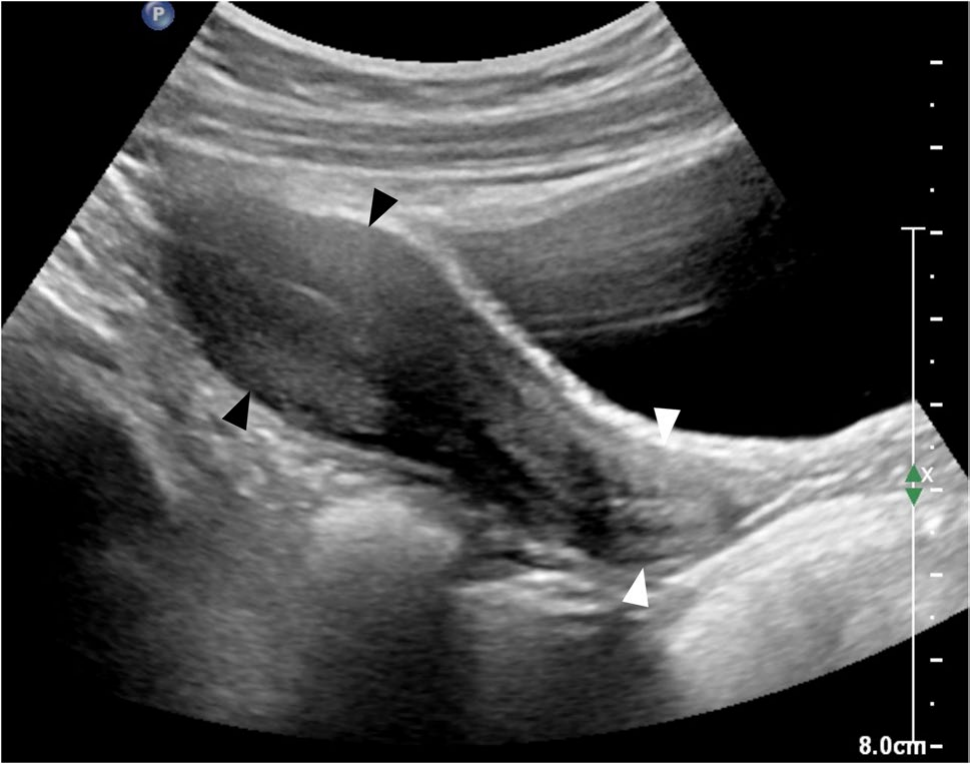
Sagittal gray-scale pelvic ultrasound image in a 13-year-old girl shows a mature pear-shaped uterus: the fundus (*black arrowheads*) is now wider than the cervix (*white arrowheads*)

At birth, the uterus is prominent with a length of about 3.5–4 cm secondary to the effect of maternal and placental sex hormones. It appears tubular or spade shaped with the anteroposterior diameter of the cervix being equal to or larger than that of the fundus (Fig. 4). As maternal and placental hormones decrease, the uterus becomes smaller and tubular with the anteroposterior diameter of the fundus equaling the cervical anteroposterior diameter and having a length of around 1–3 cm (Fig. 5) [30, 34, 35]. Somatic growth continues throughout childhood under the influence of growth hormone until the combination of pubertal sex steroids and growth hormone stimulates the uterus to grow both in length and volume [32]. The shape of the uterus then gradually changes from tubular to the adult pear-shape secondary to the increase of fundal anteroposterior diameter, leading to an increased fundus-cervix-ratio (Fig. 6).

### The endometrium

The fetal endometrium starts showing secretory changes after 33 weeks of gestation and this peaks at birth. After birth, regressional changes set in. Following a transitional phase lasting a few months, the endometrium becomes thin and remains inactive until the first signs of ovarian activity, i.e., secretion of estrogen [36].

### The ovaries

**Fig. 7:**
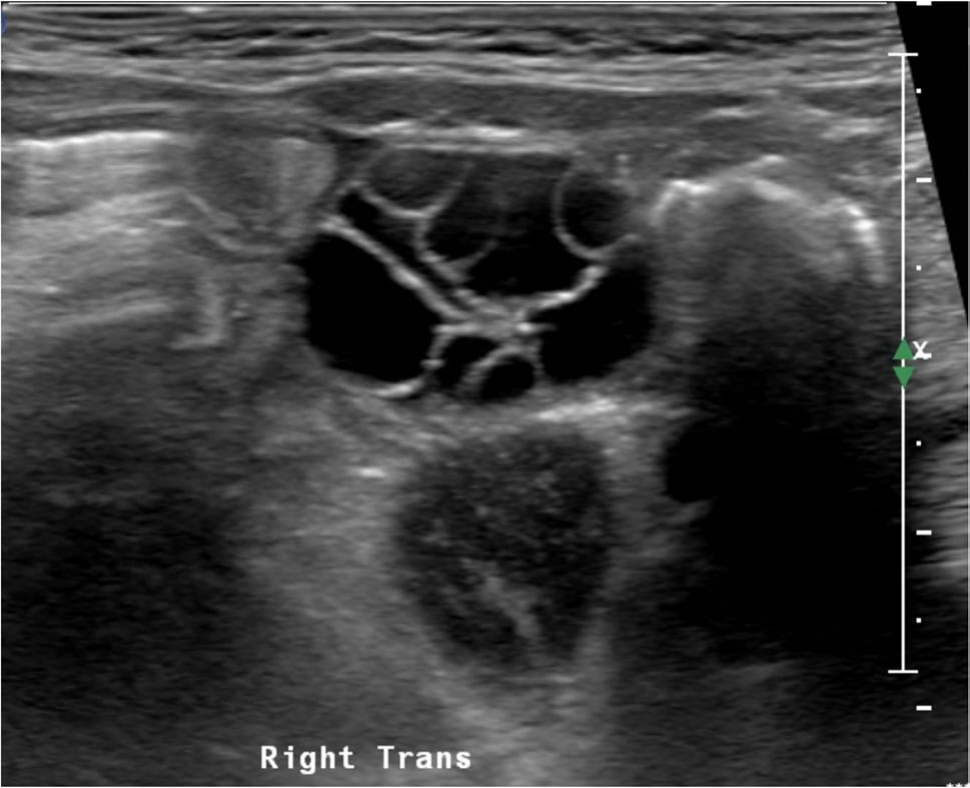
Axial gray-scale pelvic ultrasound image of a normal neonatal ovary, there are multiple follicles of different sizes

**Fig. 8:**
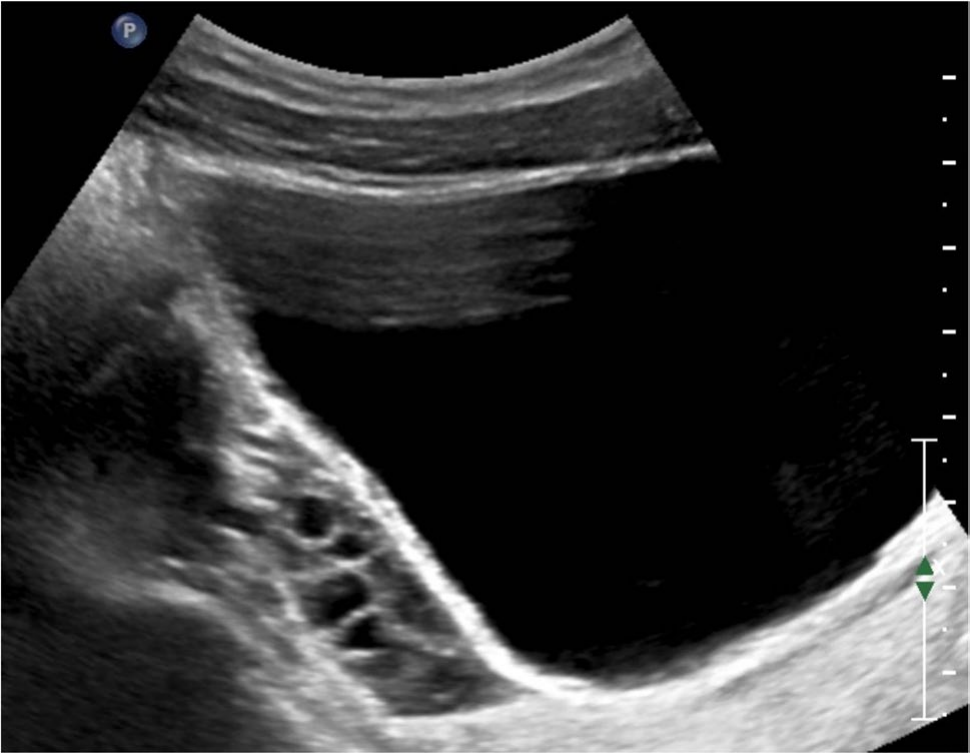
Sagittal gray-scale pelvic ultrasound image of the right ovary in an 11-year-old prepubertal girl with multiple follicles of different sizes

**Fig. 9:**
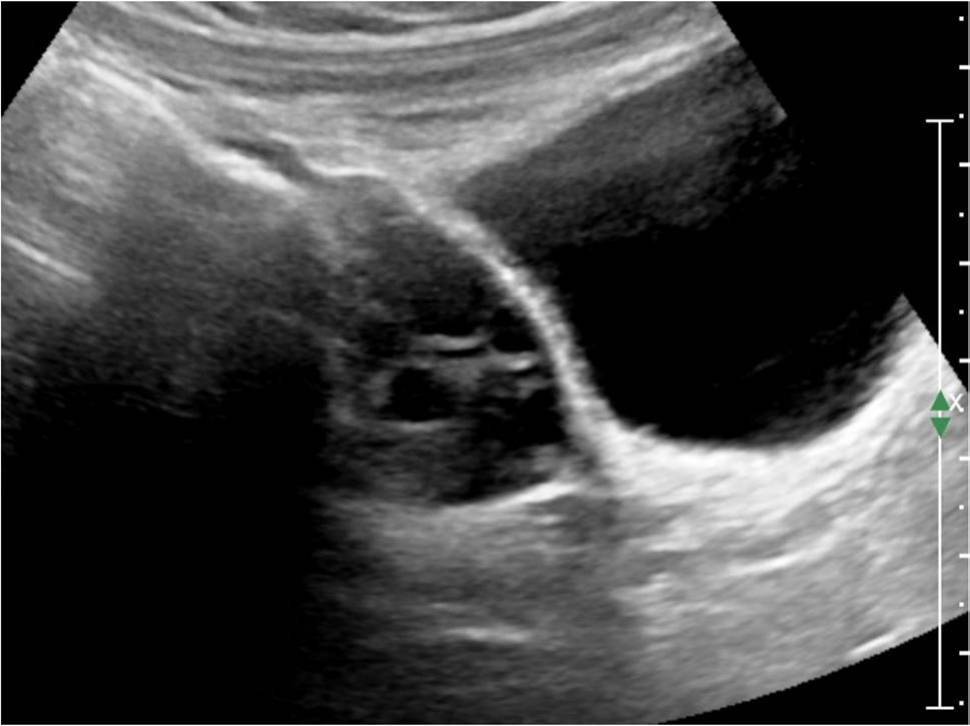
Sagittal gray-scale pelvic ultrasound image of the right ovary in a 13-year-old post-menarchal girl shows multiple follicles of different sizes

Maternal and placental hormones stimulate the ovaries. Neonatal ovaries have volumes of at least 1 ml and bear large follicles (Fig. 7). Ovaries have a stromal component that increases discreetly from birth to maturity and a follicular component dependent on FSH. Follicular activity is present in utero, during lactation and in both prepuberty and puberty when higher levels of FSH are present. Both immature and mature ovaries bear follicles in different stages of development or in atresia (Figs. 8 and 9) [37]. Both types of follicles may appear cystic [29].

### Uterine and ovarian arteries

During pubertal maturation, the vascular flow within the uterine arteries undergoes changes as the uterus grows and there is a reduction in vascular resistance concurrent with the onset of puberty. Elevated levels of LH in the bloodstream could potentially enhance vascularization [38]. Estradiol seems to reduce vascular resistance by directly affecting smooth muscle cells in the uterine artery vessel wall, or indirectly by mitigating calcium-mediated vessel constriction and/or periarterial sympathetic vasoconstrictor nerve activity [38–40]. There appears to be a notable rise in impedance towards the end of puberty, likely attributable to the culmination of uterine angiogenesis [38].

## Results of the literature review

### Study design and populations (gray-scale ultrasound)

We searched PubMed for studies on gray-scale pelvic US in central precocious puberty in girls. We selected only research papers that compared US findings in girls with central precocious puberty to US findings in girls with normal pubertal variants (premature thelarche or premature adrenarche or pubarche) and/or controls. We included 21 studies of which 12 were prospective, eight retrospective, and one cross-sectional (Table 1) [15, 24, 38, 41–58]. The age of the included children varied from 11 months to 12 years. Most studies defined precocious puberty as the development of secondary sexual characteristics before the age of 8 years. One paper defined precocious puberty as regular menses before the age of 10 years or breast budding before 8 years [47]. In all papers, the reference standard for central precocious puberty was based on the presence of premature thelarche. Additional criteria varied between the papers but all included at least one of the following criteria: increased height velocity, advanced bone age (usually by 2 years or >2 SDS but not always specified), or the pubertal response to GnRH testing.

**Table 1:** Study characteristics for studies with gray-scale ultrasound

Authors/year	Country	Ref. standard for CPP	Population	Age range
Stanhope et al. 1985 [41]	UK	Not clear	8 CPP/40 PreM	6 m–14 y
Stanhope et al. 1986 [42]	UK	Not clear	6 PP	1.4–9.3 y
Haber et al. 1995 [43]	Germany	Progressive SSC <8 y, GVA and advanced BA	20 CPP/55 PT/101 controls	2.1–7.7 y
Buzi et al. 1998 [24]	Italy	SSC <8 y GV SDS >2, BA vs CA >2y, Pos GnRH test	19 CPP/48 PT/20 PA	1.1–9.2 y
Herter et al. 2002 [44]	Brazil	Progressive SSC <8 y BA > 2 SDS, GV > 6 cm/y, Pos GnRH test	8 CPP/96 prePUB/8 PT	1–7 y
Battaglia et al. 2003 [38]	Italy	SSC <8 y (pubic hair + breast stage II), Pos GnRH test	16 PP/17 prePUB	Mean 6.3–7.2 y
De Vries et al. 2006 [15]	Israel	BB <8 y plus one or more: menses, pubic hair, GVA, or BA >2SDS	81 PP/22 PT	4–8.9 y
Badouraki et al. 2008 [45]	Greece	BB <8 years plus one or more: BA >2SDS, GV >6 cm/year, pubic/axillary hair or menses, Pos GnRH test	50 CPP/23 PT/15 PA/81 controls	2.96–8.21 y
Sathasivam et al. 2011 [46]	USA	SSC <8 years (Tanner stage II or III for breast/pubic hair), morning LH > 0.3 IU/L and estradiol >10 pg/mL, Pos GnRH	9 PP/30 prePUB/11 P	3.1–9.5 y
Eksioglu et al. 2013 [47]	Turkey	SSC <8 y or regular menstrual periods >10y or SSC >8y and BA > 2SDS and GV >2 SDS and Pos GnRH	51 CPP/56 PT/18 PA	11 m–10 y
Bizzarri et al. 2014 [48]	Italy	BB and GV >1SDS and Pos GnRH test	9 CPP/85 PT	1.56–3 y
Binay et al. 2014 [49]	Turkey	BB <8 y, BA vs CA >1 y Pos GnRH or basal LH levels > 0.3 IU/L	63 CPP/37 PT	6.72–8.6 y
Yu et al. 2015 [50]	Korea	CA 7–8 y, Tanner stage ≥2, BA vs CA >1 y, Pos GnRH	186 CPP/62 PT	7–8 y
Lee et al. 2016 [51]	Korea	BB <8 years, advanced BA, Pos GnRH	93 CPP/99 PT	Mean 7.01 and 6.62 y
Karaoglan et al. 2018 [52]	Turkey	BB <8 years plus any pubertal finding, including pos GnRH test	164 PP/103 PT	Mean 7.21 and 5.09 y
Wen et al. 2018 [53]	China	BB <8 y plus 1 or more: BA >2SDS, GV ≥6 cm/year, pubic and/or axillary hair or menses and Pos GnRH	47 CPP/59 PT or PA/25 PM	1.7–10 y
Yu et al. 2019 [54]	China	BB <8 y, plus one or more: menses, pubic hair, BA vs CA >1 year Pos GnRH	122 CPP/80 controls	6–10 y
Calcaterra et al. 2020 [55]	Italy	BB <8 y, plus one or more: menses, pubic hair, GVA, BA vs CA > 1 year, Pos GnRH-test	111 RP-CPP and 66 SP-PP	3–8 y
Yuan et al. 2021 [56]	China	BB <8 y, BB Tanner II and pos GnRH-test	350 CPP/319 PT	Median 8 and 7.5 y
Kongmanas et al. 2021 [57]	Thailand	BB < 8y and pos GnRH test	50 CPP/18 PT	2–12y
Zarei et al. 2022 [58]	Iran	SSC <8 y, BA >2 SDS and GVA and pos GnRH test	93 CPP/16 PT/62 controls	5.83–8 y

*BA* bone age, *BB* breast buds, *CA* chronological age, *CPP* central precocious puberty, *GV* growth velocity, *GVA* growth velocity acceleration,* P* pubertal, *PA* premature adrenarche, *PM* premature menarche, *preM* premenarchal, *prePUB* prepubertal, *pos GnRH-test* pubertal response to GnRH-stimulation test, *RP-CPP* rapidly progressing central precocious puberty, *SDS* standard deviation score, *SP-CPP* slowly progressing central precocious puberty, *SSC* secondary sex characteristics

### Study design and populations (Doppler ultrasound)

We searched PubMed for studies on Doppler US of uterine and/or ovarian arteries in puberty. We included only original research papers, of which seven were cross-sectional, one prospective longitudinal and one retrospective (Table 2) [38, 40, 59–65]. The populations in the studies consisted of healthy girls before, during, and after onset of puberty or patients with premature adrenarche and/or premature thelarche or patients with central precocious puberty compared to controls. The age of the included girls/women varied from 2 years to 25 years. Reference standards for precocious puberty diagnosis were Tanner breast and/or pubic hair score and/or pubertal response to GnRH testing. In one study, uterine length > 35 mm was considered one of the reference criteria for diagnosing precocious puberty [64].

**Table 2:** Study characteristics and results on measurements from color Doppler studies of uterine and ovarian arteries

Authors/year	Country	Ref. standard for CPP	Population	Age	PI index cutoffs for puberty onset
Laursen et al. 1996 [59]	Denmark	Tanner score	166 healthy volunteers 33 patients not technically suited for Doppler measurement	6–25 y	Not reported
Ziereisen et al. 2001 [60]	Belgium	Tanner score	61 healthy volunteers	2–15 y	Not reported
Battaglia et al. 2002 [61]	Italy	Tanner breast and pubic hair score and response to GnRH test	29 PT and PPU	<8 y	<2.5
Battaglia et al. 2002 [44]	Italy	Tanner breast score and basal hormonal assay	29 PPU	6.2–7.8 y	Not reported
Battaglia et al. 2003 [40]	Italy	Tanner breast and pubic hair score and response to GnRH test	69 PT and/or PPU	Mean 6.3 and 7.2 y	<2.5
Battaglia et al. 2005 [62]	Italy	Tanner and Marshall score and response to GnRH test	46 obese girls CPP (31 normal, 16 polycystic ovaries)	Mean 7.5 and 7.1 y	Not reported
Golestani et al. 2008 [63]	Iran	Tanner and Marshall score	20 prePUB, 20P, 20 post-M	Mean 8.44, 10.8, and 13.5 y	Not reported
Paesano et al. 2019 [64]	Italy	≥2 of the following: Tanner breast score ≥2, GnRH test with LH peak >5mU/mL, uterine length >35 mm	207 prePUB, 176 P, and 112 CPP	7.6 and 8.7 y	<4.6
Cheuiche et al. 2022 [65]	Brazil	Tanner score	39 prePUB, 92 initial P, 71 late P	5-16y	<5.05

*P* pubertal, *PA* premature adrenarche, *PI* pulsatility index, *post-M* postmenarchal, *PPU* premature pubarche, *prePUB* prepubertal, *PT* premature thelarche

### Gray-scale ultrasound technique

Pelvic US studies were performed or reviewed by one or more operators, (pediatric) radiologists, gynecologists, or was not specified. Scanning approach was transabdominal except for one study where the scanning approach was transrectal [56]. The transducers used were convex or linear and the frequency varied from 3 to 13.5 MHz. In all studies, scanning was obtained with a full bladder.

### Color-Doppler ultrasound technique

Doppler flow measurements of the uterine arteries were conducted transabdominally using probes with varying frequencies (3.5 MHz, 2–5 MHz, 5–8 MHz), in most cases with patients in recumbent position and employing a 50-Hz filter to eliminate low-frequency signals originating from vessel wall movements [38, 40, 59–65]. Color flow images of the ascending branches of the uterine arteries were captured laterally to the cervix in a longitudinal plane, with angle of insonation adjustment to maximize color intensity [38, 61]. Some authors performed the examinations between 08:00 and 11:00 am to mitigate the effects of circadian rhythmicity on utero-ovarian blood flow, along with a 15-min rest in a waiting room before scanning to minimize external influences on the pelvic blood flow [38, 61]. All studies were conducted using the full bladder technique [40, 59–63, 65], except for one study in which pulsatility index measurements were taken with both a full and an empty bladder [64].

### Measurements and appearance of uterus and ovaries

Uterine measurements reported included length, width, transverse area, and volume; the latter was always calculated by using the ellipsoid formula V = length × anteroposterior diameter × transverse diameter × 0.5233. In 1 study, measurements were reported separately for cervix and uterus; in the other studies, it was not reported if the cervix was included in the uterine measurements. In 13 studies, the visibility and/or thickness of the endometrium were evaluated. Only one study described the method and the location of the measurement: at its maximum anteroposterior thickness in the sagittal plane [47]. Reported ovarian measurements included height, width, and length, transverse circumference, and volume as well as appearance of the ovaries, i.e., description of solid or cystic and classification with regard to number and size of follicles: homogeneous pattern in the absence of visible follicles; microcystic: ≥1 follicle of <4 mm in diameter; paucicystic: <6 follicles with a diameter ranging from 4 mm to 9 mm; multicystic: >6 follicles with a diameter ranging from 4 to 9 mm; macrocystic: >1 follicle with a diameter larger than 9 mm; and major isolated cyst: diameter larger than 2 cm.

Reproducibility of measurements was addressed and tested in only one study [15].

Uterine and ovarian measurement cutoff values for onset of puberty proposed by the different studies can be found in Table 3. The most frequently reported uterine cutoffs are for length, volume, transverse diameter, and fundus-cervix ratio. Cutoffs for uterine volume varied from 1.07 ml to 4 ml, those for the uterine length from 22 mm to 40.9 mm, and for the uterine area from 4 cm^2^ to 4.5 cm^2^. Wen et al. reported that the cervical thickness was the best parameter for discriminating central precocious puberty from normal girls in girls between 6 years and 8 years of age [53]. Fundus-cervix ratio varied from 0.98 to 1.45. The reported ovarian volume cutoff values for the onset of puberty ranged from 1 ml to 3.5 ml.

**Table 3:** Cutoff values for gray-scale ultrasound

Authors/year	UT VOL (ml)	UT AP (mm)	UT TRS (cm)	UT L (mm)	UT area (cm^2^)	FCR	CX AP (cm)	Endom (mm)	OV VOL (ml)	OV
Stanhope et al. 1985 [41]					4				3	Megalocystic
Stanhope et al. 1986 [42]										Multicystic
Haber et al. 1995 [43]	1.8			33					1.2	
Buzi et al. 1998 [24]										
Herter et al. 2002 [44]	3			40	4.5				1	
Battaglia et al. 2003 [38]	4							Halo		
De Vries et al. 2006 [15]		> 8	1.5	34	4.5				1.96	
Badouraki et al. 2008 [45]				38.3		1.05			3.35	
Sathasivam et al. 2011 [46]										
Eksioglu et al. 2013 [47]										
Bizzarri et al. 2014 [48]										
Binay et al. 2014 [49]				30		0.98			1.3	
Yu et al. 2015 [50]	1.07			22				Presence		
Lee et al. 2016 [51]	3.3			40.9		1.45			3.5	
Karagloan et al. 2018 [52]				32					1.09	
Wen et al. 2018 [53]							0.73			
Yu et al. 2019 [54]	1.01									
Calcaterra et al. 2020 [55]			1.5	35				Presence	2	
Yuan et al. 2021 [56]				32					1.09	
Kongmanas et al. 2021 [57]	3.5		1.7						1.5	
Zarei et al. 2022 [58]	1.4							> 1		

*CX* cervix, *Endom* endometrium, *FCR* fundus-cervix-ratio, *OV VOL* ovarian volume, *UT AP* uterine anteroposterior diameter, *UT area* uterine area, *UT L* uterine length, *UT TRS* uterine transverse diameter, *UT VOL* uterine volume

The visibility of the endometrium was reported to be a discriminator for the onset of puberty in several studies. Calcaterra et al. reported that the presence of an endometrial echo is a sign of rapid progressive central precocious puberty [55]. De Vries et al. found an endometrial echo in 53% of girls with precocious puberty and in none of the girls with premature thelarche [15]. Eksioglu et al. saw the endometrial lining more frequently and with a mean thickness that was significantly higher in girls with central precocious puberty compared to premature thelarche and controls in girls between 0 years and 8 years of age [47]. Zarei et al. reported a high specificity for endometrial thickness and echogenicity for central precocious puberty [58]. In contrast, a thin endometrium was reported in some prepubertal girls by Bridges et al. [23]. Battaglia et al. reported a visible endometrial echo in girls with central precocious puberty but also less frequently in girls with premature thelarche and premature adrenarche [38]. In a recent study, Gilligan et al. reported the visibility of an endometrial stripe in all ages, with a varying thickness with age and pubertal status and Villalobos et al. detected an endometrial stripe in all healthy girls between 6 years and 12 years [28, 31]. Hagen et al. reported a considerable overlap in endometrium thickness in prepubertal and pubertal girls [66].

### Measurements from color Doppler studies of uterine and ovarian arteries

Doppler US enables the evaluation of blood flow in the utero-ovarian region, including the measurement of flow impedance through the calculation of the pulsatile index (Table 2) [40, 59–65]. The pulsatile index is defined as the difference between peak systolic flow and end-diastolic flow, divided by the mean flow velocity, and it is indicative of arterial constriction or changes in resistance to blood flow within the vascular network [67].

There have been no significant differences observed in the measured pulsatile indices of the left and right uterine arteries, leading to the utilization of the average value from both arteries or the use of data from the most easily accessible side [38, 61].

In the one study in which pulsatile index measurements were taken with both a full and an empty bladder, no differences were found [64].

Ziereisen and colleagues have categorized Doppler flow waves of the uterine arteries into three types: type 1 displayed narrow systolic flow waves without diastolic flow in the majority of prepubertal girls; type 2 exhibited systolic flow waves with interrupted signal during diastole in Tanner stage 1 and at the onset of puberty; and type 3 demonstrated broad systolic flow waves with uninterrupted diastolic flow in post-menarchal girls and in girls with Tanner breast stages 2 or 3 [60].

The threshold for uterine artery pulsatile index to detect the initiation of puberty ranged from 2.5 to 5.05 with a reported sensitivity of 77–94%, specificity of 85–100%, and accuracy of 79–97% according to various studies in the literature [67].

Recently, Paesano and colleagues reported that a combination of a pulsatile index higher than 4.6 and a longitudinal uterine diameter less than 35 mm showed an accuracy of 91% for excluding puberty onset, whereas Cheuiche et al. reported that the combination of a pulsatile index below 5.05 and a uterine volume above 3.75 cm^3^ had the highest positive predictive value (97%) for puberty onset [65].

Only one study, which assessed both ovarian and uterine Doppler US in precocious puberty, reported no correlation with pubertal stage [63].

## Discussion

Since the late 1970s, pelvic US has been explored and utilized as a tool in pediatric gynecology. The first publications on the contribution of pelvic US in the detection of idiopathic central precocious puberty appeared in the 1980s [41, 42]. Since then, both the ovarian and uterine morphology as well as different types of measurements have been evaluated as markers for onset of puberty.

We reviewed a large series of studies researching the value of pelvic US in the diagnosis of central precocious puberty. In these studies, a series of individual parameters or a combination of parameters have been evaluated. For the uterus, this included the shape or fundus-cervix ratio, the visibility of the endometrial stripe and its thickness, the length, width, area, and volume. For the ovaries, this included the volume, the transverse circumference, the visibility of follicles, the number of follicles, and the diameter of the largest follicle. What we found in this literature review is a wide variety in parameters or combinations of parameters reported as the most sensitive markers for precocious puberty as well as a wide diversity in the defined cutoff values but also four studies that concluded that pelvic US does not provide cutoffs to differentiate girls with central precocious puberty from pseudo-precocious puberty or controls [24, 46–48].

### Populations

The main populations consisted of girls with central precocious puberty; however, the reference standards for central precocious puberty that were used to compare to premature thelarche were not uniform. Moreover, none of the studies provided the duration of symptoms before being tested. Since puberty is a dynamic process, the time from onset of hormonal stimulation to the US examination should influence the measurements. It is conceivable that the “gray-zone” in the clinical presentation and the hormonal testing is reflected in the heterogeneous population and also in the heterogeneity of the results provided by pelvic US.

### Influence on measurements related to technique

There is variety in the types of transducers and frequencies used. In the most recent literature, higher frequency probes are being used, although not consistently.

All authors mention the scanning examinations being performed with a full bladder. However, overfilling may alter the measurement of the anteroposterior diameters of fundus and cervix and affect the fundus-cervix ratio [68]. In none of the studies was there any mention of this.

The repeatability of the measurements was addressed in only one study [15]. However, the precision of certain proposed cutoff values is remarkable.

Only a few studies have reported on the intra- and inter-rater reliability of arterial Doppler evaluation, with intraobserver agreement ranging between 82% and 96.7%, and a variation coefficient evaluated in only one study at 12% [64, 65].

### Uterus

We noticed a wide variety in cutoffs for the different uterine measurements. Uterine length was the most cited with a maximum cutoff of 40 mm. However, Buzi et al. found that normal prepubertal girls can have uterine lengths of 4 cm or more [24]. This is in agreement with Griffin et al. and Bridges et al. [21, 23]. In none of the studies, there was a description of how the uterine length was measured, if the cervix was included in the measurement nor if the measurement was performed with a curved or linear measurement. Volume measurement with the ellipsoid formula can be questioned since the uterus does not have an ellipsoid shape like most ovaries and because the shape of the uterus changes through puberty [21].

### Endometrium

Historically, the visibility of the endometrial stripe has been used by some to discriminate between pubertal and prepubertal state [15]. Now, several papers have reported visibility of the endometrial stripe at all ages and also a large overlap of the appearance of the endometrium between girls with central precocious puberty and healthy controls [28, 31, 61, 66]. It is imaginable that when lower frequency probes were used, the resolution might not have been adequate to visualize a thin endometrium nor small follicles in prepubertal girls. But even Bridges et al. in a study done in 1996 reported a thin endometrium in some prepubertal girls, and related it to the very low concentrations of estrogen that circulate in prepubertal girls [23]. Reina-Alzate et al. correlated imaging findings and hormonal markers at the onset of puberty in girls and found that the endometrial echo is a strong indicator of precocious puberty in girls 8 years or younger [69]. According to Wen et al., endometrial thickness of 2.6 mm is a cutoff in girls with central precocious puberty in the age group 8 years to 10 years. This is however an age at which normal puberty may set in and is not defined as central precocious puberty [53]. Moreover, no repeatability exercises were performed to justify this measurement precision.

### Ovaries

It is technically more difficult to measure the ovaries than the uterus. Angulation of the transducer through the filled bladder is often necessary, which might also affect the measurements. Some authors report that the right ovary is often easier to visualize and measure and it is often larger than the left. This could also be related to the fact that the left ovary can be (partly) obscured by gas in the sigmoid. This certainly has an impact on the reported cutoff value since many authors calculated the ovarian volume by the mean value of right and left ovary. Sathasivam et al. reported overlap in ovarian volume between girls with prepubertal and pubertal GnRH responses, regardless of the ovarian size calculation used (average, smallest, or largest) [46].

Although ovarian circumference in the transverse position rather than volume may more accurately reflect ovarian size, as ovaries, particularly in prepubertal girls, do not have a symmetrical ovoid shape, this parameter investigated by De Vries et al. and YU et al. was not a discriminator between precocious puberty and premature thelarche [15, 50, 70].

Microcysts or ovarian follicles have been reported as a normal finding in approximately 40% of prepubertal girls and De Vries et al. found no difference in number of ovarian follicles or size of the largest follicle between girls with precocious puberty and premature thelarche [15]. Millar et al. reported that observations of small, unilocular ovarian cysts of less than 1 cm in diameter in prepubertal girls are clinically unimportant but that ovarian cysts associated with precocious puberty are generally larger than 2 cm in diameter [71]. The presence of six or more follicles (multicystic ovaries) has been reported to correlate with pubertal stimuli both in premature thelarche and precocious puberty [44]. Buzi et al. found multicystic ovaries more frequently in girls with central precocious puberty than premature thelarche and controls but not exclusively [24].

### Color Doppler of uterine and ovarian arteries

Discrepancies in the reported values can be attributed to variations in the selection criteria among studies, such as whether they exclusively involve healthy girls or include those experiencing normal physiological puberty or precocious puberty. Additionally, genetic and socioeconomic differences might influence pubertal development, and variations in the reference tests used to define the onset of puberty contribute to this variability. Moreover, the outcome of US examinations is subject to operator proficiency, with the experience of the radiologist exerting a notable influence, and measuring uterine artery velocity becomes increasingly difficult with decreasing age [59, 67].

### Existing guidelines

The European Society of Paediatric Radiology has not published guidelines regarding the role of imaging in the workup of precocious puberty, and to our knowledge, there are no other unifying international guidelines. National guidelines differ from each other. For example, in the Netherlands, there are no strict guidelines regarding workup for central precocious puberty and pelvic US is not included as a mandatory test in the recommendations. Some national guidelines recommend pelvic US in the workup of precocious puberty, both to detect possible underlying causes of precocious puberty but also to evaluate the internal genitalia to aid in the diagnosis of central precocious puberty but indicate no cutoff for the measurements (e.g., Norway). Yet, other national guidelines provide cutoff values for uterine size and/or endometrium and/or ovaries; however, the cutoff values vary somewhat between guidelines (e.g., Italian, Greek). A review paper from 2023 on the diagnosis, treatment, and outcomes in central precocious puberty, published in Lancet Child Adolescent Health, states that US cannot diagnose onset of puberty due to the overlap of size measurements in girls with central precocious puberty and normal girls, that US may have a role in equivocal cases, and that uterine length over 3.5–4 cm and a volume over 3 mL support the diagnosis. However, neither the sensitivity and specificity of these values nor their positive and negative predictive values when applied to this specific population are mentioned [72].

There is a striking lack of consistent guidelines for US imaging in the workup of central precocious puberty despite the noninvasive, accessible nature of this technique. We believe this reflects the inhomogeneity of the literature in the field as described in this paper.

Ideally, future studies should be harmonized in terms of populations included, definitions of central precocious puberty, and technique applied. In addition, repeatability exercises should be performed to determine the reasonable precision of the various measures. However, central precocious puberty is a dynamic condition and the precise onset of symptoms, hence also disease duration, is difficult to determine. Further, genetic and environmental factors may influence both time of onset and course of the puberty process. A single US examination only represents a “snapshot” in this variable and individual process. Therefore, it might be difficult to harmonize the populations to find exact cutoff values for onset of puberty with US.

## Conclusions

A wide variety of thresholds have been reported regarding measurements of uterus and ovaries. The endometrium can be seen as an echogenic stripe and ovarian follicles and cysts can be seen in both prepubertal and pubertal girls.

Although there is a clear role for abdominal US in peripheral precocious puberty, we have to be aware of the limitations of pelvic US in the diagnosis of central precocious puberty. Interpretation of the findings must be done with caution, particularly in equivocal cases, and close communication with the referring clinicians is crucial. No reliable cutoffs exist to determine onset of puberty in girls. This should be reflected in relevant guidelines.
